# Aberrant resting-state functional connectivity of the dorsolateral prefrontal cortex to the anterior insula and its association with fear avoidance belief in chronic neck pain patients

**DOI:** 10.1371/journal.pone.0221023

**Published:** 2019-08-12

**Authors:** Naho Ihara, Kenta Wakaizumi, Daisuke Nishimura, Jungo Kato, Takashige Yamada, Takeshi Suzuki, Saori Hashiguchi, Yuri Terasawa, Shizuko Kosugi, Hiroshi Morisaki

**Affiliations:** 1 Department of Anesthesiology, Keio University School of Medicine, Tokyo, Japan; 2 Department of Psychology, Keio University, Tokyo, Japan; Florida State University, UNITED STATES

## Abstract

Chronic neck pain (CNP), a global health problem, involves a large amount of psychological and socioeconomic burdens. Not only physical causes but also behavioral disorders such as a fear-avoidance belief (FAB) can associate with the chronicity of neck pain. However, functional brain mechanisms underlying CNP and its related behavioral disorders remain unknown. The aim of the current resting-state functional magnetic resonance imaging (fMRI) study was to explore how the functional brain networks differed between CNP patients and age- and sex-matched healthy, pain-free controls (HCs). We also investigated whether these possible brain network changes in CNP patients were associated with fear avoidance belief (FAB) and the intensity of pain. We analyzed the resting-state fMRI data of 20 CNP patients and 20 HCs. FAB and the intensity of pain were assessed by Tampa Scale for Kinesiophobia (TSK) and Visual Analog Scale (VAS) of pain. The whole brain analysis showed that CNP patients had significant different functional connectivity (FC) compared with HCs, and the right dorsolateral prefrontal cortex (DLPFC) was a core hub of these altered functional networks. Furthermore, general linear model analyses showed that, in CNP patients, the increased FC between the right DLPFC and the right anterior insular cortex (aIC) significantly associated with increased TSK (p = 0.01, statistical significance after Bonferroni correction: p<0.025), and the FC between the right DLPFC and dorsal posterior cingulate cortex had a trend of inverse association with VAS (p = 0.04). Our findings suggest that aberrant FCs between the right DLPFC and aIC associated with CNP and its related FAB.

## Introduction

Chronic neck pain (CNP), a global health problem, poses a large socioeconomic burden, including restrictions on social life, reduced work productivity, and the cost of healthcare. The global point prevalence of cervical pain was reported to be about 5%, and the large population of patients suffer neck pain-related disability.[1, 2]

Numerous neuroimaging studies involving other musculoskeletal pain such as chronic low back pain (CLBP) have shown widespread structural and functional brain changes in individuals with chronic pain compared with healthy, pain-free controls (HCs).[3, 4] These changes may be associated with emotional, cognitive and behavioral properties of chronic pain patients.[5–7] Resting-state functional magnetic resonance imaging (fMRI) studies have shown altered functional connectivity (FC) in three main networks: the central executive network (CEN) (dorsolateral prefrontal cortex [DLPFC] and posterior parietal cortex), the salience network (SN) (anterior insular cortices [aIC] and anterior cingulate cortex [ACC]), and the default mode network (DMN) (posterior cingulate cortex [PCC]/precuneus[Pcu], medial prefrontal cortex [mPFC], and angular gyrus) in patients with CLBP and knee osteoarthritis (OA).[8–11]

On the other hand, despite the large population of patients, neuroimaging analyses in CNP are still understudied. Neck pain often results in physical disorders in the upper part of the body and is accompanied by symptoms such as headache and shoulder stiffness.[12] These unique properties may present with a different type of discomfort and disability from that experienced in CLBP and OA, possibly leading to CNP-specific alteration in the functional brain network. Two resting-state fMRI studies showed significantly different brain activity in specific brain areas such as middle frontal gyrus, left insula, and superior frontal gyrus in CNP patients compared to control groups.[13, 14] However, functional brain networks in CNP remain unknown. Furthermore, CNP is attributed to not only biological causes but also behavioral disorders, such as pain catastrophizing and fear avoidance beliefs (FAB).[15–17] A fear avoidance model involving these psychological and behavioral disorders is broadly accepted to explain a vicious cycle of persistent pain.[18] In fact, FAB has been reported to associate with the prevalence and prognosis of chronic musculoskeletal pain including neck pain.[16, 17, 19–21] Several neuroimaging studies of CLBP indicated that behavioral components included in fear avoidance model such as pain catastrophizing and FAB may be associated with the disrupted functional brain network.[5, 7, 22] However, the underlying brain mechanisms of FAB in CNP is also unknown.

We hypothesized that CNP patients would show different functional brain networks compared to individuals without pain in resting state. We also hypothesized that these disturbed networks would be associated with increased pain and FAB in CNP patients. For this purpose, we explored whether and how the resting-state FCs in CNP patients differed compared to HCs. In addition, we investigated whether these possible FC differences in CNP patients were associated with FAB and the severity of pain.

## Materials and methods

### Ethics statement

This study was approved by the institutional ethics committee of Keio University School of Medicine (authorization number: 20160002), and all subjects provided their written informed consent in line with the Declaration of Helsinki. The study was registered in the University Hospital Medical Information Network Clinical Trials Registry (ID: 000024475).

### Subjects and clinical assessment measures

Twenty-one right-handed CNP patients from outpatients of Keio University Hospital and 25 age- and sex- matched HCs were enrolled. One CNP patient and 5 HCs were excluded due to a high rate of invalid volumes, as mentioned below. A total of 20 CNP patients and 20 HCs were analyzed in this study.

Specific inclusion criteria for chronic neck pain were 1) experiencing pain at the neck, 2) pain persisting for three months or longer, and 3) pain of ≥4 on the Numerical Rating Scale (NRS; scale of 0–10) at the initial visit. Subjects with any psychiatric disorders or any brain anatomical abnormalities and ones who were expected to have difficulty keeping their head in the same position because of neck pain during fMRI scanning were excluded from the enrollment of the study.

We measured the current pain on a Visual Analog Scale (VAS; 0–100 mm) and FAB on the short-version of Tampa Scale for Kinesiophobia (TSK; 11–44 points).[23, 24] Subjects answered these questionnaires on the same day as their fMRI examination.

### Data acquisition and preprocessing

A 3.0-T Sigma MRI system (GE Healthcare, Chicago, IL, USA) with an 8-channel phased array coil was used to obtain brain imaging data. Anatomical images were acquired with 3-dimensional brain volume imaging with an extended dynamic range T1-weighted sequence: voxel size = 1 × 1 × 1 mm; inversion time (TI)/flip angle (FA) = 650 ms/8°; field of view (FOV) = 256 mm.

Resting state functional images were acquired using gradient echo planner imaging: sequence = ascending; slice thickness/gap = 3.2/0.8 mm; FOV = 212 mm; acquisition matrix = 64 × 64 mm, repetition time (TR) = 2500 ms; echo time (TE) = 30 ms; and flip angle = 80°; number of slices = 40; resting scanning time = 8 min. All subjects were directed to stay awake with their eyes open without thinking of any specific thing during scanning. To confirm subjects’ state of awakeness during scanning, we performed the Stanford Sleepiness Scale (SSS; scale of 1–7)[25] immediately after scanning. We regarded SSS ≥ 5 as an exclusion criterion for data analysis, but no one met this criterion.[26]

The fMRI data were preprocessed using the CONN toolbox Version 17.f, [27] running on MATLAB version R2015b (Mathworks, Inc., Natick, MA, USA). The preprocessing encompassed discarding the first four volumes to allow for magnetic field stabilization, skull extraction, slice time correction, motion correction, high-pass and low-pass temporal filtering (0.008 and 0.09 Hz), and scrubbing outlier volumes identified by Artifact Detection Toolbox. Several sources of noise that might contribute to non-neuronal fluctuations were removed from the data through linear regression. These included the six parameters obtained by rigid body correction of head motion, the global blood oxygenation level dependent (BOLD) signal averaged over all voxels of the brain, and the signals from five components each of the white matter and CSF region. Subjects with a high rate of outlier volumes (>10%) were excluded from the analysis. Overall, all included healthy controls and patients exhibited minimal artifacts (standardized global brain activation < 5 and relative mean displacement < 1 mm). There was no significant difference in the number of volumes remained after the scrubbing process between the groups. All preprocessed data were then normalized spatially in accordance with the Montreal Neurological Institute (MNI) template. Finally, all resulting images were smoothed isotropic a Gaussian filter kernel, with a full width at half maximum of 5 mm.

### Statistical analyses

#### Questionnaire data analysis

The normality of the questionnaire data was assessed using the Shapiro-Wilk test. The clinical assessment measures were compared between the CNP and HC groups using Wilcoxon’s rank test with the JMP software program (ver.13.0.0; Cary, NC, USA).

#### Functional connectivity analysis

**Fig 1:**
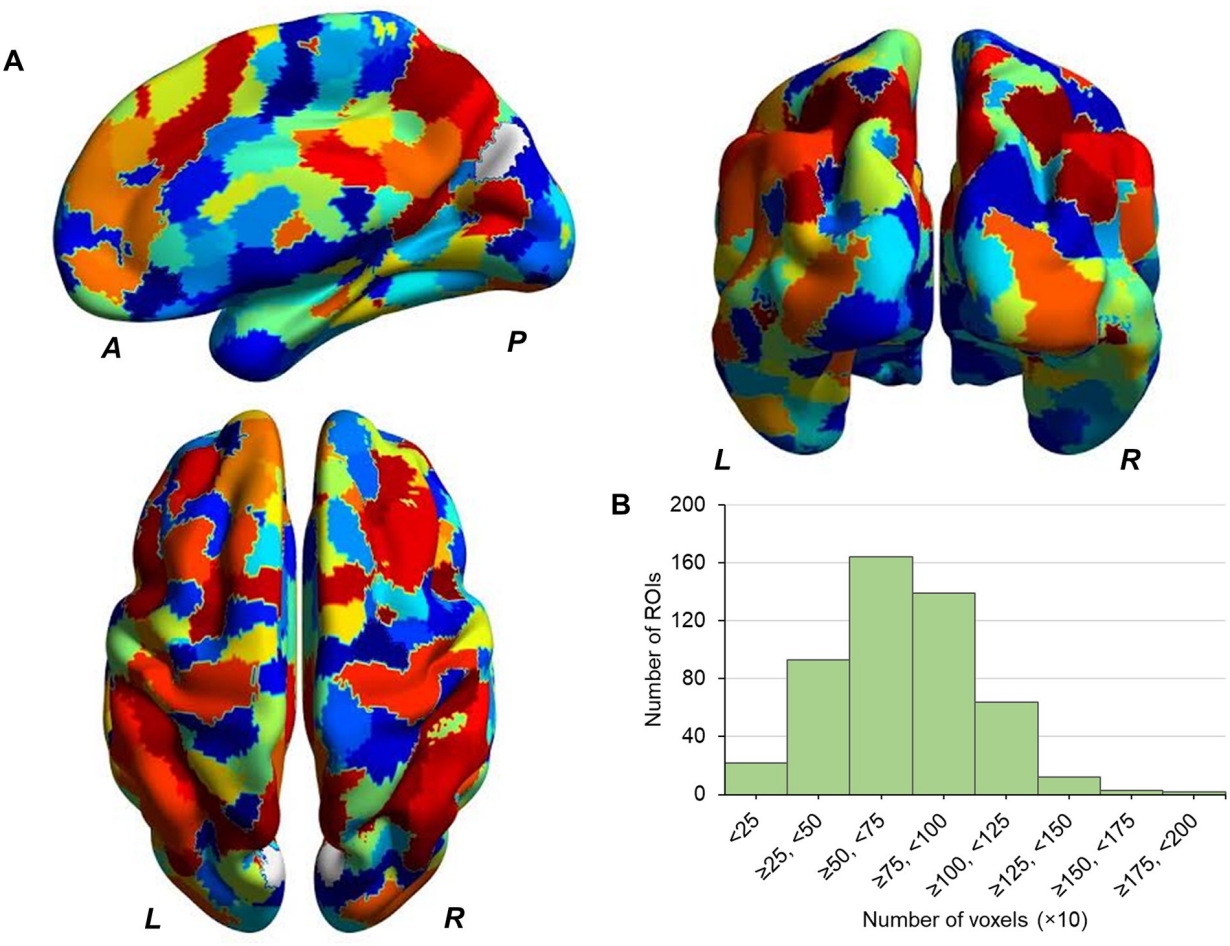
Brain surface schema of 499 regions. (A) Brain surface schema of 499 regions. (B) Histogram showing a distribution of number of voxels each region contains. L: left, R: right, A: anterior, P: posterior, ROI: region of interest.

Each voxel was assigned to 499 well-validated regions of interest (ROIs) for each participant (Fig 1). The parcellation of 499 ROIs was derived from functional brain signals using a Ward hierarchical clustering incorporated with connectivity constraints, respecting to common functional networks identified by an independent component analysis.[28] Optimal parcellation was determined by supervised clustering using Python Scikit-learn.[29] BOLD signal of ROI was computed as an average across voxels contained in each ROI.

CONN calculated the Pearson’s correlation coefficients (r-values) of time courses of BOLD signals for all combinations of ROIs and then analyzed the FC using the Fisher’s transformed r-values. A general linear model (GLM) analysis and false discovery rate (FDR)-corrected p-values (p-FDR) < 0.05 were used to identify the significance of the whole-brain analysis for group-level comparisons after adjusting age and sex. The brain network results were visualized using the BrainNet Viewer (http://www.nitrc.org/projects/bnv/).[30]

#### Association between clinical factors and functional connectivity

In the whole brain analysis, the right DLPFC had 15 FCs that were significantly different between CNP patients and HCs (p-FDR<0.05), thereby was identified as a ROI with the largest number of significant links. Moreover, these significant DLPFC FCs included ROIs in the SN and DMN which had the extensive a priori evidence regarding the involvement in chronic pain. Therefore, we regarded the right DLPFC as an important hub in the functional brain networks in CNP, and focused on the association between these 15 DLPFC FCs and clinical assessment measures (TSK and VAS). To identify this association, additional GLM analyses on TSK and VAS were performed specifying these DLPFC FCs as covariates of interest using the JMP software program (ver.13.0.0; Cary, NC, USA). To determine the final GLMs, these 15 DLPFC FCs were examined in a stepwise backward and forward selection procedure using the criterion of P <0.1. After this selection process, we also considered the rationality of a parameter estimate of each FC retained in stepwise selection procedure. Namely, as for FCs that exhibited CNP>HC in the whole brain analysis, we considered a FC with positive parameter estimate in clinical association analysis in CNP patients to be a rational covariate to the model. Conversely, as for FCs that exhibited CNP<HC, we considered a FC with negative parameter estimate in clinical association analysis in patients to be rational. Accordingly, when the positive/negative of the estimate of each FCs retained in stepwise analysis contradicted the group-level contrast in the whole brain analysis, such FCs were excluded from the final model. The final GLMs of TSK and VAS were constructed by including age, sex, and the FCs selected by the process described above. For these two GLM analyses, Bonferroni correction for multiple testing was applied (statistical significance; p<0.05/2).

#### Post-hoc gray matter volume analysis

Gray matter volume (GMV) based on a T1-weighted structural image was preprocessed including skull stripping, gray and white matter segmentation, B1 bias field collection, non-linear registration to an MNI space, and surface labeling corresponding to a predetermined anatomical brain atlas [31] using FreeSurfer v6.0 (http://surfer.nmr.mgh.harvard.edu/), an open-source software validated previously.[32–35] We chose anatomical parcels including the DLPFC, the aIC, and the dorsal PCC identified in the functional analyses and applied age- and sex-adjusted GLM to examine differences of gray matter volumes between HC and CNP and association of the volumes with TSK and VAS in CNP.

## Results

### Demographic and clinical assessment measures

Demographic data and questionnaire scores are shown in Table 1. The TSK scores were significantly higher in the CNP group than in the HC group.

**Table 1:** Demographic and questionnaire scores.

	CNP (n = 20)	HC (n = 20)	p value ^a^
Age, y	45.2 ± 10.3	44.5 ± 9.9	0.75
Male, n (%)	6 (30)	6 (30)	-
Disease duration, month	47.2 ± 40.7	-	-
VAS	49.9 ± 21.6	-	-
TSK	27.2 ± 4.3	16.8 ± 5.4	< 0.01

^a^: Wilcoxon’s rank test.

data are presented as mean ± standard deviation values or as the number of patients (percentage).

CNP: chronic neck pain, HC: healthy control, VAS: Visual Analog Scale, TSK: Tampa Scale for Kinesiophobia.

### Whole-brain analyses for group-level comparisons

**Fig 2:**
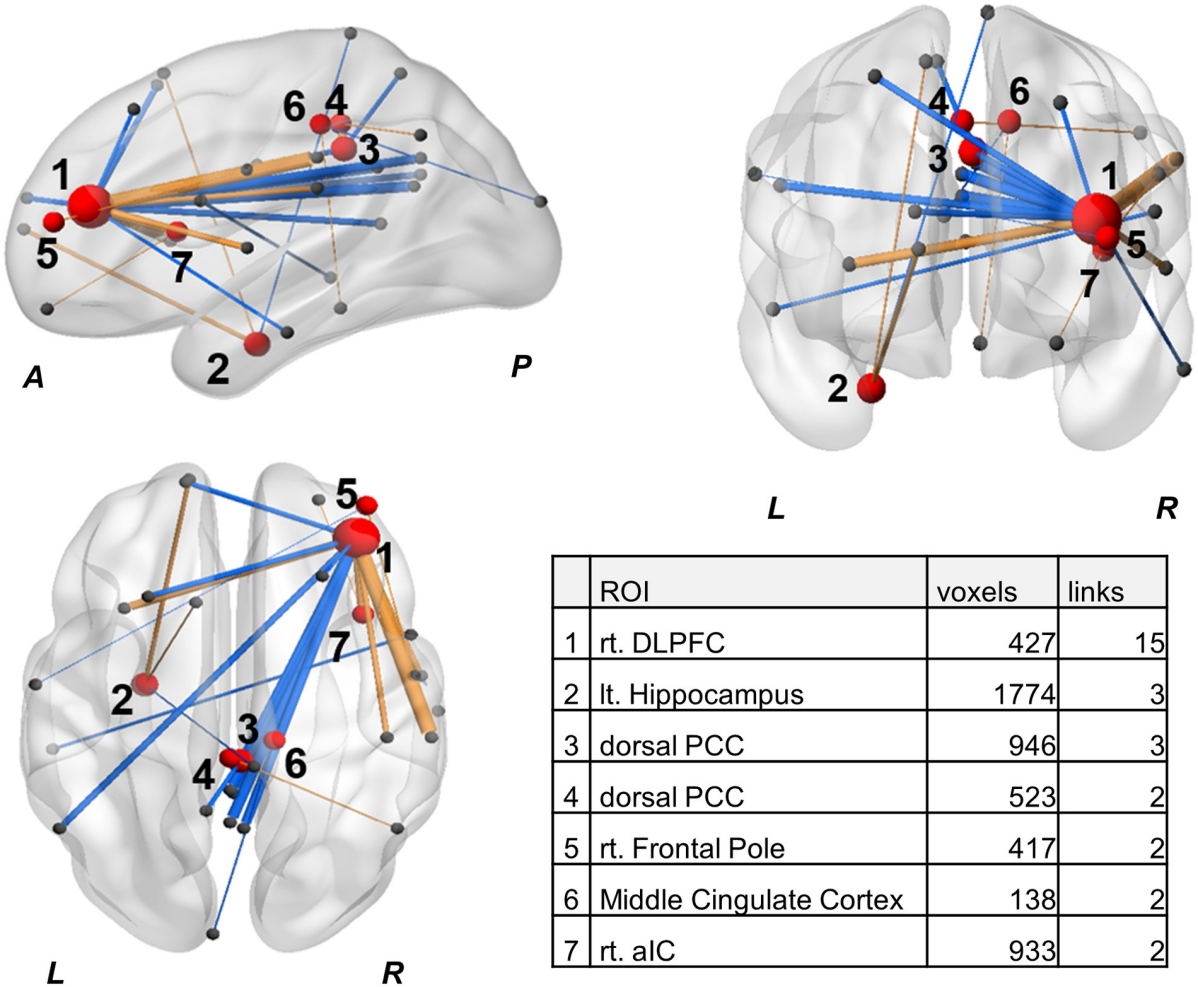
Whole brain functional connectivity map for group-level comparison (CNP vs. HC). ROIs with two or more significant different links in group comparison were shown as red spheres. Size of spheres represents number of links (p-FDR<0.05) that each ROI has. Width and color of links represent the correlation coefficient and the vector of each connection; CNP>HC; orange, CNP<HC; blue. The numbers of voxels and links of each ROI were shown in the table in the figure. HC: healthy control, CNP: chronic neck pain, ROI: region of interest, p-FDR: false discovery rate-corrected p-values, FC: functional connectivity, L: left, R: right, A: anterior, P: posterior, rt: right, lt: left, DLPFC: dorsolateral prefrontal cortex, PCC: posterior cingulate cortex, aIC: anterior insular cortex.

We identified 29 connections of 38 ROIs with significantly different FCs between the CNP patients and HCs (Table 2). PCC subregions were identified according to Vogt’s description.[36] ROIs with multidirectional connectivity changes, i.e. with two or more significant different links in group comparison, were shown in Fig 2. A ROI with the largest number of significant links was identified as the right DLPFC according to the 499 ROI atlas.

**Table 2:** Functional Connectivity with significant difference between CNP and HC on the whole brain analysis.

ROI (MNI Coordinates)	ROI	MNI coordinates	T-values	p-FDR
CNP>HC				
rt. DLPFC (36, 40, 18)	rt. Central Opercular Cortex	55, -7, 5	3.50	0.012
	rt. Parietal Operculum Cortex	45, -28, 24	3.57	0.024
	rt. aIC	38, 14, 10	3.70	0.028
	rt. Supramarginal Gyrus	58, -28, 34	3.96	0.028
	lt. aIC	-32, 16, 6	4.26	0.028
lt. hippocampus (26, -10, -27)	lt. Frontal Pole	-13, 60, 10	4.03	0.046
	lt. Superior Frontal Gyrus	-11, 18, 60	4.33	0.046
rt. Frontal Pole (39, 51, 13)	lt. Postcentral Gyrus	-58, -10, 30	4.27	0.034
	rt. Precingulate Gyrus	53, -7, 30	4.46	0.034
rt. aIC (38, 14, 10)	rt. Frontal Pole	25, 53, -15	4.46	0.038
rt. Cuneal Cortex (3, -88, 37)	rt. Superior Parietal Lobule	27, -42, 70	4.45	0.040
CNP<HC				
rt. DLPFC (36, 40, 18)	dorsal PCC	2, -36, 36	4.61	0.012
	rt. Middle Temporal Gyrus	60, -19, -22	4.35	0.024
	rt. Pcu	3, -59, 33	3.94	0.028
	ventral PCC	-1, -46, 30	3.94	0.028
	ventral PCC	0, -47, 12	3.86	0.028
	lt. Angular Gyrus	-51, -59, 27	3.60	0.047
	lt. DLPFC	-14, 59, 20	3.49	0.048
	lt. Superior Frontal Gyrus	-25, 20, 56	3.46	0.048
	Pcu	-1, -57, 24	3.45	0.048
	rt. Middle Frontal Gyrus	26, 27, 49	4.06	0.028
lt. hippocampus (26, -10, -27)	Post Postcentral Gyrus	1, -38, 77	4.05	0.046
dorsal PCC (2, -36, 36)	lt. Pcu	-8, -53, 60	4.23	0.026
dorsal PCC (-1, -35, 44)	dorsal PCC	2, -36, 36	4.96	0.009
	rt. Lateral Occipital Cortex	48, -59, 41	4.22	0.039
Middle Cingulate Cortex (12, -29, 44)	lt. Occipital Pole	-6, -95, 19	4.92	0.009
	brain stem	4, -35, -15	4.28	0.033
lt. Middle Temporal Gyrus (-53, -32, -6)	rt. Precingulate Gyrus	52, 7, 20	5.59	0.001
lt. Paracingulate Gyrus (-1, 54, 9)	rt. Superior Parietal Lobule	23, -53, 57	4.50	0.034

MNI coordinates (x, y, z) are shown in mm.

ROI: Region of interest, CNP: chronic neck pain, HC: healthy control, p-FDR: false discovery rate-corrected p-values, rt: right, lt: left, DLPFC: dorsolateral prefrontal cortex, aIC: anterior insular cortex, PCC: posterior cingulate cortex, Pcu: Precuneus Cortex.

### Association between clinical factors and functional connectivity

**Fig 3:**
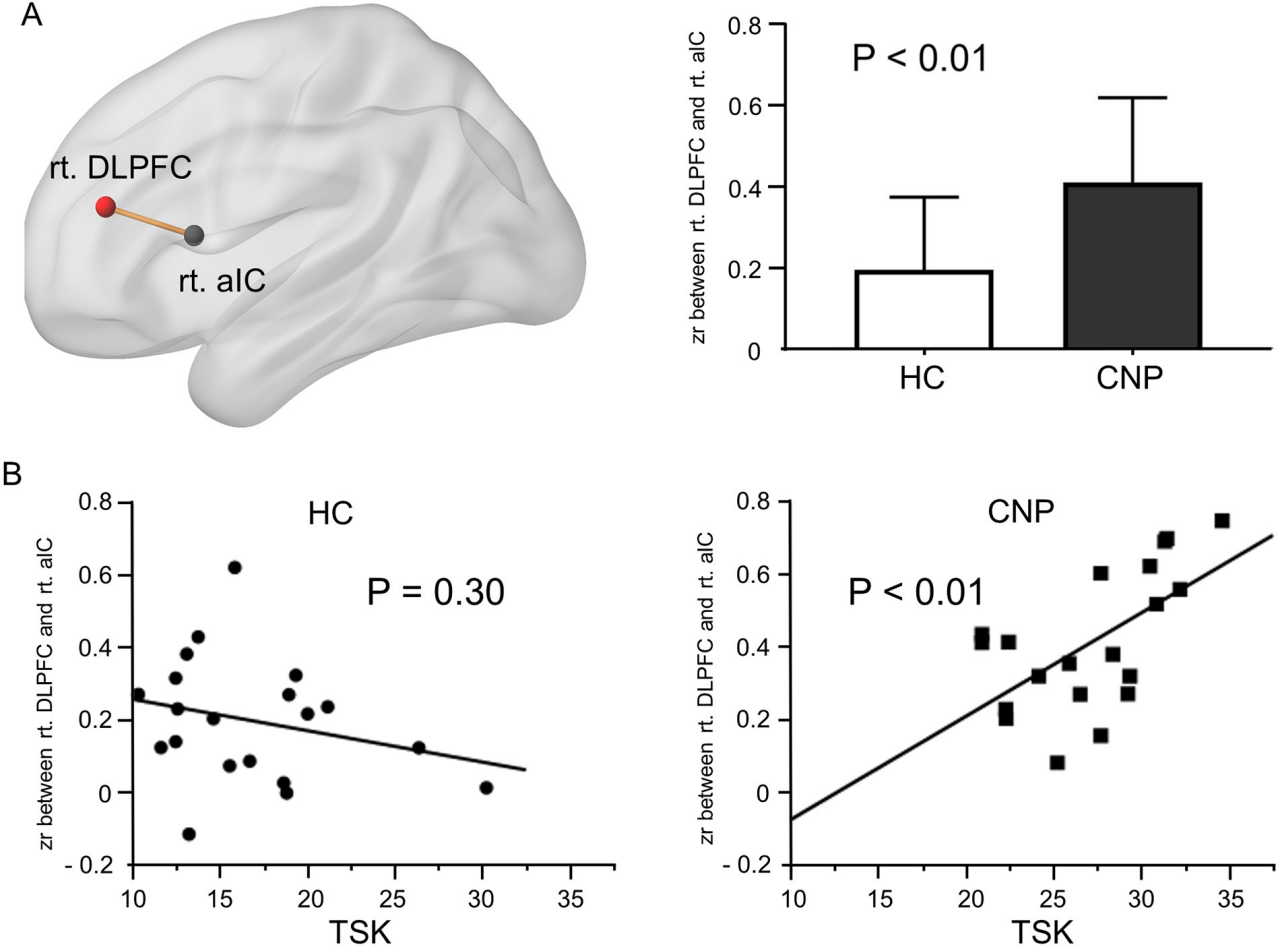
Brain network associated with TSK. **(A)** Group difference in functional connectivity (zr) between rt. DLPFC and rt. aIC. Data are expressed as mean and standard deviation. (B) Association between the TSK and FC (rt. DLPFC-rt. aIC) in HC and CNP. Residuals added to means of each score were plotted under age- and sex-adjusted regression analyses. P values represent significance of partial regression coefficient. rt: right, DLPFC: dorsolateral prefrontal cortex, aIC: anterior insular cortex, FC: functional connectivity, HC: healthy control, CNP: chronic neck pain, TSK: Tampa Scale of Kinesiophobia, zr: Fisher’s transformed correlation coefficient.

After a stepwise selection and a consideration of rationality of each parameter estimate, the FCs of the right DLPFC to the right aIC and the right Pcu were retained as significant determinants for TSK, and FCs to the dorsal PCC and the right supramarginal gyrus were retained for VAS. Thus, the final GLMs were constructed for the association of these FCs with TSK and VAS (Table 3). The GLM for TSK revealed that increased FC between the right DLPFC and the right aIC was significantly associated with increased TSK in CNP patients. The GLM for VAS showed a trend of inverse association between VAS and the intensity of FC between the right DLPFC and dorsal PCC, whereas the association was insignificant after Bonferroni correction. Group difference in FC between the right DFPLC and the right aIC and the association between TSK and the FC in CNP were shown in Fig 3A and 3B. For reference, we examined the association of the FC between right DLPFC and right aIC with TSK in HCs, whereas no significant association was observed. (Fig 3B).

**Table 3:** The final GLMs for association between clinical factors and functional connectivity of the right DLPFC in CNP patients (N = 20). Age- and sex-adjusted.

Clinical factors	FC	β	T-value	P-value
VAS	adjusted R2 = 0.39, F = 4.01, p = 0.02			
	dorsal PCC	-0.42	-2.25	0.04
	rt. Supramarginal Gyrus	0.41	2.01	0.06
TSK	adjusted R2 = 0.31, F = 3.09, p = 0.04			
	rt. aIC	0.60	2.84	0.01
	rt. Pcu	-0.05	-0.25	0.8

GLM: general linear model, CNP: chronic neck pain, FC: functional connectivity, VAS: visual analog scale, TSK: Tampa Scale for Kinesiophobia, rt: right, DLPFC: dorsolateral prefrontal cortex, PCC: posterior cingulate cortex, aIC: anterior insular cortex, Pcu: Precuneus Cortex.

### Post-hoc gray matter volume analysis

The dorsal PCC showed significantly lager GMV in CNP patients than in HC. No significant difference was found in GMV of the right DLPFC and the right aIC between the groups. There were no significant associations of the GMVs of these regions with TSK and VAS in CNP (Table 4).

**Table 4:** Group differences in gray matter volume of the main regions and correlations with clinical variables in CNP patients. Age- and sex adjusted.

ROI	Gray matter volume		Correlation with clinical variables in CNP (N = 20)			
	CNP>HC		VAS		TSK	
	T-value	P-value	T-value	P-value	T-value	P-value
rt DLPFC	0.75	0.46	-1.66	0.12	-1.12	0.28
rt aIC	-0.15	0.88	-0.84	0.49	0,19	0.85
dorsal PCC	2.69	0.01	1.56	0.13	0.61	0.55

ROI: Region of interest, CNP: chronic neck pain, HC: healthy control, rt: right, DLPFC: dorsolateral prefrontal cortex, PCC: posterior cingulate cortex, aIC: anterior insular cortex, VAS: visual analog scale, TSK: Tampa Scale for Kinesiophobia.

## Discussion

Our data showed that, in the resting state, CNP patients had significant different functional brain networks compared with HCs, and the right DLPFC was a core hub of these altered functional networks. We also showed that the FC between the right DLPFC and the right aIC significantly associated with patients’ kinesiophobia, suggesting that aberrant right DLPFC connectivity to the right aIC may play a key role in FAB in CNP patients.

### The altered functional connectivity in chronic neck pain

The state of the brain in chronic pain encompasses both the disruption of whole-brain network and the disruption of local FCs. As a disorganized whole brain network is commonly observed in various chronic pain conditions, more generalizable neuroimaging analyses viewing large-scale brain functional reorganization have been proposed rather than analyses focusing on a specific local brain region.[37] However, as the disruption of local brain functional network and its clinical association are unique to the types of the chronic pain, [8–11] the accumulation of neuroimaging data focusing on local brain regions may also help understanding of disease-specific alteration in local functional connections. To our knowledge, this is the first study to show CNP-specific change in FC and its association with FAB and pain. From our results of the whole-brain analysis of 499 ROIs, similar to other musculoskeletal pain such as CLBP and OA, CNP patients also showed several altered FCs of the CEN, SN, and DMN. A systematic review of neuroimaging studies on CLBP summarized that the altered DMN activity and/or connectivity were predominantly associated with CLBP.[4] Several resting state fMRI studies showed increased DMN FCs to aIC, ACC, and inferior parietal lobule and decreased FC between mPFC and precuneus in CLBP, [9, 11] and increased anticorrelation between the aIC and DMN and decreased FC between mPFC and precuneus in OA patients, [9, 10] indicating that the SN and DMN are key networks in chronic musculoskeletal pain. Interestingly, in our study, the DLPFC, a hub of the CEN, had the largest number of links with different FC between CNP patients and HCs. Futhermore, these included links to aIC (SN) and PCC and precuneus (DMN). This specific finding suggests that aberrant FC of the DLPFC may be strongly associated with neural mechanisms of the chronicity of neck pain.

### The role of DLPFC in pain

The DLPFC has been shown to be mainly related to cognitive processes such as attention, decision-making, working memory, planning ability, learning ability, and regulating emotion. [38–41] The DLPFC is also included in the nociceptive system, which recognizes and processes pain both perceptually (primary and secondary somatosensory cortex and thalamus) and affectively (ACC, DLPFC, and aIC). [42]

Experimental pain stimuli evoke bilateral DLPFC activation [43–45] and enhance the DLPFC connectivity to other brain regions, such as the aIC and ACC.[46] Furthermore, the activation of the DLPFC has been suggested to be involved in pain suppression. [45, 47–49] On the other hand, in chronic pain patients, some studies have found that the gray matter volume or density in the DLPFC was reduced compared to HCs, and these structural abnormalities were associated with pain.[50–52] In addition, other resting-state and task-based fMRI studies have indicated that abnormal activation or connectivity of DLPFC in chronic pain patients was restored by pain treatment or placebo.[53–55]. These findings imply that structural and functional abnormalities of the DLPFC may dull pain suppression, consequently leading to the augmentation and prolongation of pain, but these abnormalities of the DLPFC may be reversible by proper pain treatments.

Our results showed decreased FCs between the right DLPFC and PCC subregions in CNP patients compared to HCs. Furthermore, the FC between the right DLPFC and dorsal PCC had a trend of inverse correlation with the intensity of pain. The PCC, a key hub of the DMN, is another important region in pain modulation. [4, 56] In a previous task-based fMRI study, PCC was activated during pain stimulation. This stimulus-driven activation of PCC was augmented in CLBP patients compared to HCs.[57] Another fMRI study showed dorsal PCC activation during task evoking pain catastrophizing was positively correlated with chronic pain intensity in fibromyalgia patients.[58] These evidence suggest that the PCC, specifically, the dorsal subregion is related to increasing pain perception. Taken together, a decreased FC between the DLPFC and dorsal PCC in CNP may be related to disrupting normal pain suppression processing, consequently increasing pain intensity.

### Chronic neck pain and fear avoidance belief

According to previous studies, FAB is significantly stronger in chronic pain patients than in others, [19, 20] and the intensity of FAB was a significant predictor of recovery from impairment.[17, 59] Thus, the FAB is a core factor contributing to the chronicity of musculoskeletal pain, and modification of the FAB in chronic patients can be an important treatment target to facilitate an early recovery from pain. Identifying brain regions and their functional connection associated with the FAB of chronic pain patients may aid in visualizing these therapeutic effects.

The TSK, which was originally developed for the evaluation of FAB in low back pain patients, has also been validated for pain in other parts of the body, including neck pain. The TSK score has been suggested to be a sensitive predictor of the duration and degree of pain-related disability and resistance of treatment in neck pain patients.[60–62] A previous study showed that neurokinin 1 receptor function in the right ventromedial prefrontal cortex (VMPFC) was associated with TSK. [63] This finding can infer that the function of the VMPFC and its involving brain networks could be associated with FAB in chronic pain patients. From our additional voxel-wise analysis performed by placing a seed on the VMPFC, we found some significant FCs in the group-level comparison, while these FCs were not associated with TSK in CNP patients (data not shown). Interestingly, our results showed a significant association between TSK and the DLPFC connectivity in CNP patients, i.e., increased FC between the right DLPFC and the right aIC was significantly associated with greater TSK scores, while no significant association between the FC of these ROIs and TSK was observed in HCs.

The aICs have abundant connections to other brain regions and play a key role in integrating perceptual, cognitive, and affective pain.[44, 64, 65] A task-based fMRI study reported that the TSK correlated positively with the activation of the aIC; furthermore, in CLBP, the TSK correlated differently with the FC between aIC and the amygdala compared with HCs.[22]

While few studies to date have described the association of the DLPFC with FAB, the DLPFC activity has been reported to have negative correlations with pain unpleasantness.[45] Furthermore, a task-based fMRI study showed that pain catastrophizing, another element of the fear avoidance model, correlated negatively with the DLPFC activity but positively with the IC activity evoked by pain stimuli.[66] A resting-state fMRI study showed that pain catastrophizing negatively correlated with the FC between the DLPFC and PCC in HCs but positively in chronic pain patients.[67] Taken together, these findings suggest that the DLPFC activity and its connection to other brain regions, such as the aIC and PCC, may play a key role in modulating pain-related negative feelings in HCs, but such functional integration between these brain regions may be disrupted in chronic pain patients, resulting in the augmentation of fear avoidance and catastrophizing beliefs.

### Brain structural implication in chronic neck pain

A structural brain change is another important aspect to understanding central mechanism underlying chronic pain. Structural MRI studies have shown gray matter abnormalities in the regions such as the DLPFC, IC, ACC, and PCC in chronic pain. [54, 67, 68] While we focused on the functional brain networks in CNP throughout the study, we performed an additional structural brain analysis to explore the potential involvement of structural brain change in the observed FC differences between groups. Although our functional analysis showed that the right DLPFC and the right aIC were the key regions associated with patients’ behaviors, neither structural differences between patients and controls nor their associations with patients’ behaviors were found. On the other hand, an increased GMV of the dorsal PCC was observed in CNP patients while this structural change was not directly correlated with patients’ behaviors. Such a structural change in the dorsal PCC may involve in observed FC differences between groups, while the relationship between the structural and functional brain changes is still unknown, and each of these brain changes may provide unique information on pain cognition and behavior.[68]

### Limitations

Several limitations associated with the present study warrant mention. First, most patients had used various medications, such as pregabalin, tramadol, and/or antidepressants, to relieve pain, so we were unable to exclude the possible effect of these medication on the brain functions. Second, no significant correlation was found between the TSK and the intensity of pain, suggesting that FAB may estimate chronicity of neck pain via the disruption of functional brain network, but not the degree of individual pain. More complex functional changes in the brain may associate with the augmentation of neck pain. Third, because of the cross-sectional study design, we were unable to demonstrate the causal relationship of FAB, pain, and the altered FCs of brain regions. Therefore, whether or not the altered FCs in the brain may affect FAB and thereby lead to the chronicity of pain remains unclear. To clarify these causal relationships, sequential data of neuroimaging and clinical evaluations from the onset of pain are required.

## Conclusions

The alteration of DLPFC connectivity may be involved in the pathology of CNP. Furthermore, an aberrant FC between the DLPFC and aIC may disrupt normal integration in processing the FAB in CNP patients. Therapeutic strategies amending FAB may restore normal brain functional network, consequently help stop the vicious cycle of CNP. Further prospective longitudinal and interventional studies are necessary to clarify these aspects.

## Supporting information

S1 File Raw data used for all analyses. (XLSX)
